# Eliminating Phase Drift for Distributed Optical Fiber Acoustic Sensing System with Empirical Mode Decomposition

**DOI:** 10.3390/s19245392

**Published:** 2019-12-06

**Authors:** Yuejuan Lv, Pengfei Wang, Yu Wang, Xin Liu, Qing Bai, Peihong Li, Hongjuan Zhang, Yan Gao, Baoquan Jin

**Affiliations:** 1 Key Laboratory of Advanced Transducers and Intelligent Control Systems (Ministry of Education and Shanxi Province), College of Physics and Optoelectronics, Taiyuan University of Technology, Taiyuan 030024, Shanxi, China; jxcbh2@163.com (Y.L.); wangyu@tyut.edu.cn (Y.W.); liuxin0924@link.tyut.edu.cn (X.L.); baiqing@tyut.edu.cn (Q.B.); lipeihong0581@link.tyut.edu.cn (P.L.); 2 College of Electrical and Power Engineering, Taiyuan University of Technology, Taiyuan 030024, Shanxi, China; w159753wpf@163.com (P.W.); zhanghongjuan@tyut.edu.cn (H.Z.); gaoyan@tyut.edu.cn (Y.G.); 3 State Key Laboratory of Coal and CBM Co-mining, Jincheng 048000, Shanxi, China

**Keywords:** phase-sensitive optical time domain reflectometry, distributed acoustic sensing system, phase drift elimination, empirical mode decomposition, phase recovery

## Abstract

Phase-drift elimination is crucial to vibration recovery in the coherent detection phase-sensitive optical time domain reflectometry system. The phase drift drives the whole phase signal fluctuation as a baseline, and its negative effect is obvious when the detection time is long. In this paper, empirical mode decomposition (EMD) is presented to extract and eliminate the phase drift adaptively. It decomposes the signal by utilizing the characteristic time scale of the data, and the baseline is eventually obtained. It is validated by theory and experiment that the phase drift deteriorates seriously when the length of the vibration region increases. In an experiment, the phase drift was eliminated under the conditions of different vibration frequencies of 1 Hz, 5 Hz, and 10 Hz. The phase drift was also eliminated with different vibration intensities. Furthermore, the linear relationship between phase and vibration intensity is demonstrated with a correlation coefficient of 99.99%. The vibrations at 0.5 Hz and 0.3 Hz were detected with signal-to-noise ratios (SNRs) of 55.58 dB and 64.44 dB. With this method, when the vibration frequency is at the level of Hz or sub-Hz, the phase drift can be eliminated. This contributes to the detection and recovery of low-frequency perturbation events in practical applications.

## 1. Introduction 

A distributed optical fiber sensor has the characteristics of a simple structure, anti-electromagnetic interference, and intrinsic safety. Thus, it has good application prospects in the fields of structure health monitoring, perimeter security and so on [1,2,3]. As a representative sensor type among many distributed optical fiber sensors, phase-sensitive optical time-domain reflectometry (Ф-OTDR) can simultaneously detect and locate multiple external disturbances by demodulating the intensity change of a Rayleigh backscattered (RBS) signal. The superior properties of the Ф-OTDR system include high sensitivity, high spatial resolution, and fast response speed [4,5,6]. The Ф-OTDR system has broad application fields in long-distance oil and gas pipelines, mining safety, and border security intrusion detection [7,8,9]. For the Φ-OTDR system, amplitude detection could only qualitatively reflect the vibration signal, while phase demodulation could achieve the quantitative measurement and directly represent the vibration signal [10,11].

In order to recover the vibration phase, the coherent detection Ф-OTDR system is presented. The coherent detection Ф-OTDR system utilizes the quadrature demodulation and unwrapping algorithm to realize amplitude and phase demodulation at the same time, and then recovers the acoustic signal [12,13,14]. There are many serious problems in the demodulation process of an acoustic signal, such as the laser-frequency drift (LFD) and accumulative phase noise. Usually, the coherent detection Ф-OTDR system requires a stable, ultra-narrow linewidth light source. Nevertheless, most common commercial lasers do not have a stable frequency and have the problem of low LFD. Accumulative phase noise is produced during light propagation along the sensing fiber. It can be compensated by calculating the phase difference between two locations on the fiber, but some noise still exists [15]. Both LFD and accumulative phase noise drive the whole phase signal drift up and down and cause the signal to be asymmetrically distributed about the *Y* axis. Due to their same suppression effect on phase recovery, they are collectively called phase drift. In general, it is reported that the detectable frequency range of the Ф-OTDR is from Hz to MHz [16]. For certain application situations such as earthquake field monitoring and power cable galloping in the smart grid, the disturbance frequency is approximately at the level of Hz or sub-Hz [17]. When low-frequency vibration occurs, a long detection time is needed to restore the phase. Because the phase drift slowly varies with time and is seen as a baseline, the negative effect will be obvious under these conditions. In addition, when the frequency of external perturbation is at the level of sub-Hz, the phase drift will mix with vibration in the current acquisition system. For these two reasons, low-frequency vibration events could not be accurately restored using existing methods. Thus, many studies have been carried out in recent years to solve this noticeable problem. However, most research has been aimed at the LFD problem, and few studies have been carried out on the issue of accumulative phase noise [18,19].

The existence of frequency drift with a common commercial laser source was verified, and an active compensation method for the Ф-OTDR system was proposed [20]. This method replaced the anamorphic RBS trace by a new RBS trace which has the most similar frequency point in the closest sweep cycle, and then the negative effect produced by LFD on phase demodulation was offset. Besides this, the RBS traces were analyzed by cross-correlation technology, and then the amplitude and direction of the LFD were obtained in this way. The combination of frequency sweep and cross-correlation tracked and took out the LFD noise. It was validated that the phase difference in two undisturbed regions can be used for compensation of the frequency deviation in the RBS traces [21]. This method has resulted in an enhancement of the signal-to-noise ratio (SNR) up to 17 dB. The problems of time-skew and phase-mismatch were solved by utilizing a 90° optical hybrid during the process of phase demodulation [22]. The system structure was changed by combining a weak reflector array with the coherent detection Ф-OTDR system, and then the compensation of the phase noise was realized [23]. This method is only suitable for the quasi-distributed optical fiber-sensing system. The influence of LFD was compensated for using a twice differential method, and a 0.1 Hz vibration detection and restoration on a 6 km sensing fiber was finally reached [18]. In addition, the slow variation process of a stepper motor was successfully monitored and recovered. 

The elimination of phase drift is an issue that must to be solved urgently because it severely affects the recovery quality of acoustic signals and the monitoring of low-frequency disturbance events. The phase drift is usually regarded as a floor noise that can be extracted from the phase result after demodulation. The vibration signal has the characteristics of non-stationarity and rapid change. Due to the requirement of extracting and eliminating phase drift and the properties of the vibration signal, an innovative algorithm needs to be presented to eliminate the severely negative effect of phase drift on phase demodulation in the coherent detection Ф-OTDR system. 

In this paper, in order to eliminate the negative influence of phase drift in the coherent detection Ф-OTDR system, an empirical mode decomposition (EMD) method is presented. This novel method can extract the phase-drift noise and real vibration phase from the phase signal after demodulation. Furthermore, the relationship between phase drift and the length of the vibration region is also identified via theory and experiments. With the proposed method, the phase drift can be eliminated, even if the vibration is a low-frequency sinusoidal signal (sub-Hz frequency). In addition, the linear correlation between the phase and vibration intensity is also proved when the intensity of the vibration signal changes. In conclusion, the existence of phase drift and the feasibility and effectiveness of the EMD method are proved successfully from many aspects in the coherent detection Ф-OTDR system.

## 2. Principle and Methods

### 2.1. The Experimental Set-Up of the Phase-Sensitive Optical Time-Domain Reflectometry (Ф-OTDR) System

**Figure 1:**
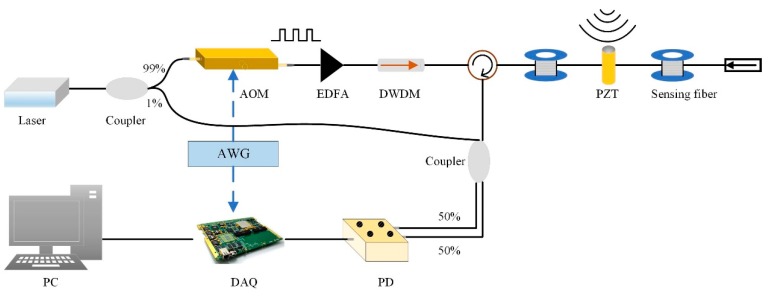
The experimental set-up of the phase-sensitive optical time-domain reflectometry (Ф-OTDR) system.

The experimental set-up of the Ф-OTDR system is shown in Figure 1. The benefit of the optical path structure is that it is simple and no additional devices are added. The ultra-narrow linewidth laser is divided into two paths with a 99:1 coupler. The 1% light is regarded as a local oscillator light, and the 99% light is modulated into pulsed light by an acousto-optic modulator (AOM). The pulsed light is injected into an erbium-doped fiber amplifier (EDFA) to amplify the light power. Then, the pulsed light goes through a dense wavelength division multiplexer (DWDM), which can filter out the light of other wavelengths except 1550 nm, and then is launched into the sensing fiber via an optical circulator (OC). A piezoelectric transducer (PZT) is used to simulate the regular vibration signal. Afterwards, the RBS light, which is the mutual interference result of numerous scatters within the pulse duration, is returned. The local oscillator light and RBS light are fully mixed by a 2 × 2 coupler. The beat signal is produced in the bandwidth-limited photodetector (PD). Then, the beat signal is transformed into the electric signal. Eventually, the signal is acquired by the data acquisition card (DAQ) and processed on the host computer. The modulation of the pulse light and the trigger of the DAQ are realized by an arbitrary waveform generator. 

### 2.2. The Generation Mechanism of Phase Drift

The phase information directly represents the external disturbance, so the phase information can be demodulated to obtain the vibration signal. Phase information can be obtained by quadrature demodulation of the beat signal. However, the phase after demodulation is induced not only by external disturbance but also by the low-frequency phase drift. The phase drift will mix with the actual phase produced by external vibration and affect the demodulation result. So, in order to accurately achieve restoration of low-frequency vibration events, the problem of phase drift must be solved. The generation mechanism of phase drift is studied in this section.

**Figure 2:**
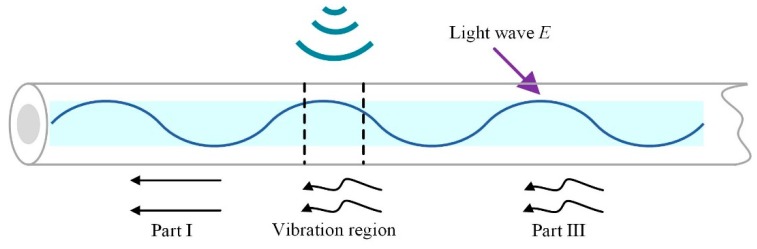
The generation mechanism model of Rayleigh backscattering trace.

Commonly, the RBS trace is generated within the injected pulse duration when the pulsed light propagates along the optical fiber. The generation mechanism model of the RBS trace is shown in Figure 2 [24]. Within the duration of half the pulse width, the accumulative light field of the RBS is expressed as Equation (1) [25]:

(1) $$E=E_{0}\overset{P+Q}{\underset{i=P}{\sum }}e_{i}\;exp\left(-2\alpha s_{i}\right)\;exp\left(j\left(\varphi_{i}-ks_{i}+2\pi f\left(t\right)t\right)\right)$$

where *E*_0_ is the initial amplitude of the injected pulsed light; *P* denotes the first scattering center; *Q* is the number of the scattering centers within the half pulse width; *i* is the label of each scattering center; *e*_i_ and *ϕ*_i_ are the amplitude and phase of the *i-th* scattering center, and the value of *ϕ*_i_ varies with position; *s*_i_ is the position of the *i-th* scattering center within the half pulse width; *k* is the wavenumber of the pulsed light; *f*(*t*) is the frequency of the pulsed light, which is the sum of the laser frequency and the frequency shift of the AOM, denoted *f*_L1_*(t)* and *f*_AOM_, respectively. Generally, the laser frequency is constant. However, the frequency value changes with a slow trend for commercial laser sources in practical applications.

The area of half pulse width is divided into three sections—part I, the vibration region, and part III—according to the effect produced by the disturbance on the sensing fiber. Equation (1) can be decomposed into three parts and thus transformed into Equation (2):

(2) $$\begin{array}{l} E=E_{0}\overset{P+Q_{1}}{\underset{i=P}{\sum }}e_{i}\;exp\left(-2\alpha s_{i}\right)\;exp\left(j\left(\varphi_{i}-ks_{i}+2\pi f\left(t\right)t\right)\right)\\ \;+E_{0}\overset{P+Q_{1}+Q_{2}}{\underset{i=P+Q_{1}+1}{\sum }}e_{i}\;exp\left(-2\alpha s_{i}\right)\;exp\left(j\left(\varphi_{i}-ks_{i}+2\pi f\left(t\right)t\right)\right)\\ \;+E_{0}\overset{P+Q_{1}+Q_{2}+Q_{3}}{\underset{i=P+Q_{1}+Q_{2}+1}{\sum }}e_{i}\;exp\left(-2\alpha s_{i}\right)\;exp\left(j\left(\varphi_{i}-ks_{i}+2\pi f\left(t\right)t\right)\right) \end{array}$$

where *Q*_1_, *Q*_2_, and *Q*_3_ are the numbers of the scattering centers in part I, the vibration region and part III, respectively. Because the length of half pulse width is usually short, the effect induced by light attenuation is tiny and can be neglected. Thus, the attenuation coefficient (*α*) can be ignored in Equation (2). In addition, compared with the size of half pulse width, the action range caused by vibration is assumed to be a point or small range. Thus, the value of Q_2_ is far smaller than the value of Q_1_ or Q_3_. The contribution of the vibration region on the whole light field is so tiny that it can be neglected. Equation (3) is obtained by making these adjustments:

(3) $$E=E_{0}\sum_{i=P}^{P+Q_{1}}e_{i}\;exp\left(j\left(\varphi_{i}-ks_{i}+2\pi f\left(t\right)t\right)\right)+E_{0}\sum_{i=P+Q_{1}+Q_{2}+1}^{P+Q_{1}+Q_{2}+Q_{3}}e_{i}\;exp\left(j\left(\varphi_{i}-ks_{i}+2\pi f\left(t\right)t\right)\right)$$

When the external perturbance is exerted on the sensing fiber, the refractive index of the fiber will change and the light phase will be modulated. Part I is, thereby, not affected, while part III experiences double phase modulation. The double phase modulation is the accumulation of the distributed phase change in part III and is denoted Δ*ϕ*. Therefore, the light field is described by Equation (4) when the external disturbance occurs.

(4) $$E=E_{0}\sum_{i=P}^{P+Q_{1}}e_{i}\;exp\left(j\left(\varphi_{i}-ks_{i}+2\pi f\left(t\right)t\right)\right)+E_{0}\sum_{i=P+Q_{1}+Q_{2}+1}^{P+Q_{1}+Q_{2}+Q_{3}}e_{i}\;exp\left(j\left(\varphi_{i}-ks_{i}+2\pi f\left(t\right)t+\Delta \varphi \right)\right)$$

By simplification, Equation (4) can be further expressed as:

(5) $$E=E_{1}\left(t\right)\;exp\left(j\left(\theta_{1}\left(s,t\right)+2\pi f\left(t\right)t\right)\right)+E_{2}\left(t\right)\;exp\left(j\left(\theta_{2}\left(s,t\right)+2\pi f\left(t\right)t+\Delta \varphi \right)\right)$$

where

(6) $$\begin{array}{c} E_{1}\left(t\right)=E_{0}{\left[{\left(\overset{P+Q_{1}}{\underset{i=P}{\sum }}e_{i}\;cos\left(\varphi_{i}-ks_{i}\right)\right)}^{2}+{\left(\overset{P+Q_{1}}{\underset{i=P}{\sum }}e_{i}\;sin\left(\varphi_{i}-ks_{i}\right)\right)}^{2}\right]}^{\frac{1}{2}}\\ E_{2}\left(t\right)=E_{0}{\left[{\left(\overset{P+Q_{1}+Q_{2}+Q_{3}}{\underset{i=P+Q_{1}+Q_{2}+1}{\sum }}e_{i}\;cos\left(\varphi_{i}-ks_{i}\right)\right)}^{2}+{\left(\overset{P+Q_{1}+Q_{2}+Q_{3}}{\underset{i=P+Q_{1}+Q_{2}+1}{\sum }}e_{i}\;sin\left(\varphi_{i}-ks_{i}\right)\right)}^{2}\right]}^{\frac{1}{2}} \end{array}$$

(7) $$\begin{array}{c} \theta_{1}\left(s,t\right)=tg^{-1}\left(\frac{\sum_{i=P}^{P+Q_{1}}e_{i}\;sin\left(\varphi_{i}-ks_{i}\right)}{\sum_{i=P}^{P+Q_{1}}e_{i}\;cos\left(\varphi_{i}-ks_{i}\right)}\right)\\ \theta_{2}\left(s,t\right)=tg^{-1}\left(\frac{\sum_{i=P+Q_{1}+Q_{2}+1}^{P+Q_{1}+Q_{2}+Q_{3}}e_{i}\;sin\left(\varphi_{i}-ks_{i}\right)}{\sum_{i=P+Q_{1}+Q_{2}+1}^{P+Q_{1}+Q_{2}+Q_{3}}e_{i}\;cos\left(\varphi_{i}-ks_{i}\right)}\right) \end{array}$$

Here, *E*_1_(*t*) and *E*_2_(*t*) are the light field intensity values of part I and part III, respectively, while *θ*_1_(*s,t*) and *θ*_2_(*s,t*) are the accumulative phase values of part I and part III, which are related to position *s*. From Equations (6) and (7), the values of *ϕ*_i_ and *k* are stochastic constants in ideal conditions, but they vary with time due to the existence of inevitable frequency drift and the different scatter positions. For convenience of description and comprehension, the light field of the RBS signal is expressed as Equation (8):

(8) $$E_{RBS}=A\left(t\right)\;exp\left(j\left(2\pi f\left(t\right)t+\Delta \phi \left(t\right)\right)\right)$$

where

(9) $$\begin{array}{c} A\left(t\right)={\left(E_{1}^{2}\left(t\right)+E_{2}^{2}\left(t\right)+2E_{1}\left(t\right)E_{2}\left(t\right)\;cos\left(\theta_{2}\left(s,t\right)-\theta_{1}\left(s,t\right)+\Delta \varphi \right)\right)}^{\frac{1}{2}}\\ \Delta \phi \left(t\right)=\frac{1}{2}\left(\theta_{2}\left(s,t\right)+\theta_{1}\left(s,t\right)+\Delta \varphi \right) \end{array}$$

Similarly, the light field of the local oscillator light is described using Equation (10):

(10) $$E_{LO}=A_{LO}\left(t\right)\;exp\left(j\left(2\pi f_{L}{}_{2}\left(t\right)t+\varphi_{LO}\right)\right)$$

where *f*_L2_(*t*) and *ϕ*_LO_ express the frequency and initial phase of the local oscillator light, respectively.

The beat signal can be obtained by coherent detection, which is the mutual interference result between the local oscillator light and the RBS light in the Ф-OTDR system. Then, the beat signal can be captured by the bandwidth-limited PD and transformed into an electric signal. The electric signal is filtered through the bandpass filter and is then described by Equation (11):

(11) $$\begin{array}{c} I\left(s,t\right)\propto A\left(t\right)A_{LO}\left(t\right)\;cos\left[2\pi \left(f_{L1}\left(t\right)-f_{L2}\left(t\right)\right)t+2\pi f_{AOM}t+\Delta \phi \left(t\right)-\varphi_{LO}\right]\\ =A\left(t\right)A_{LO}\left(t\right)\;cos\left[\frac{4\pi ns}{c}\Delta f\left(t\right)+2\pi f_{AOM}t+\Delta \phi \left(t\right)-\varphi_{LO}\right] \end{array}$$

where *n* is the refractive index, and *c* is the velocity of light waves in a vacuum. In general, the laser frequency is seen as unchanged, while the LFD exists for many laser sources. That is to say that the LFD is not equal to zero and is denoted Δ*f*(*t*). In order to recover the external perturbation, the phase induced by vibration must be obtained, which can be extracted by the quadrature demodulation and unwrapping algorithm. The phase variation after demodulation is shown in Equation (12):

(12) $$\Phi \left(s,t\right)=\frac{4\pi ns}{c}\Delta f\left(t\right)+\Delta \phi \left(t\right)-\varphi_{LO}=\frac{4\pi ns}{c}\Delta f\left(t\right)+\frac{1}{2}\theta_{2}\left(s,t\right)+\frac{1}{2}\theta_{1}\left(s,t\right)+\frac{1}{2}\Delta \varphi -\varphi_{LO}$$

Suppose that *a* and *b* are two positions with an interval of Δ*s*, and then the *Ф*(*s*_a_*,t*) and *Ф*(*s*_b_*,t*) are obtained. By calculating the difference value between them, the common part is removed and the difference component is left, as shown in Equation (13):

(13) $$\begin{array}{c} \Delta \Phi \left(\Delta s,t\right)=\frac{4\pi n\Delta s}{c}\Delta f\left(t\right)+\frac{1}{2}\left(\theta_{2}\left(s_{a},t\right)-\theta_{2}\left(s_{b},t\right)\right)+\frac{1}{2}\left(\theta_{1}\left(s_{a},t\right)-\theta_{1}\left(s_{b},t\right)\right)+\frac{1}{2}\Delta \varphi =\\ \frac{4\pi n\Delta s}{c}\Delta f\left(t\right)+\Delta \theta \left(\Delta s,t\right)+\frac{1}{2}\Delta \varphi \end{array}$$

where $\Delta \theta \left(\Delta s,t\right)=\frac{1}{2}\left(\theta_{2}\left(s_{a},t\right)-\theta_{2}\left(s_{b},t\right)+\theta_{1}\left(s_{a},t\right)-\theta_{1}\left(s_{b},t\right)\right)$. This represents the phase difference between two positions in the Ф-OTDR system, and it will deteriorate when the Δ*s* increases. From Equation (13), we can see that Δ*Ф*(Δ*s,t*) is related not only to external vibration but also to the LFD and phase accumulation at different positions. The value of Δ*s* is usually not equal to zero and expresses the length of the vibration region. Therefore, Δ*θ*(Δ*s,t*) will always exist and is correlated with the value of Δ*s*. So, it will have an obvious negative influence on phase demodulation. In addition, the LFD also always exists and is a low-frequency change. The phase variation which is caused by LFD is great and varies with Δ*s*. The combination of phase change produced by the LFD and accumulation phase is called phase drift, which is denoted *ϕ*_drift_(Δ*s,t*), and the value of *ϕ*_drift_(Δ*s,t*) is related to Δ*s*. It is worth noting that Δ*s* is correlated with the spatial resolution and the length of the vibration region in the Ф-OTDR system. Therefore, in order to obtain the actual phase variation which is produced by external disturbance, the elimination of phase drift is essential and extremely significant.

If the optical fiber is placed in a changing temperature field, the change in temperature will introduce phase (*∆φ*_T_) to the fiber. Alternatively, when the fiber is subjected to external strain, the phase (*∆φ*_strain_) of the optical fiber also changes. These changes can be described as follows:

(14) $$\begin{array}{l} \frac{\Delta \varphi_{T}}{\Delta T}=k_{0}\left(L\frac{dn}{dT}+n\frac{dL}{dT}\right)\\ \Delta \varphi_{strain}=\beta \Delta L+L\Delta \beta =\beta L\left(\frac{\Delta L}{L}\right)+L\left(\frac{\partial \beta }{\partial n}\right)\Delta n \end{array}$$

where *k*_0_ is the wavenumber of light in a vacuum; *L* is the fiber length; *n* expresses the refractive index; *T* is the temperature; *β* is the propagation coefficient in the fiber; *∆L* represents the length change of the fiber, and *∆n* denotes the refractive index change. The temperature variation causes changes in the refractive index and fiber length. From Equation (14), the phase (*∆φ*_T_) produced by temperature change varies with *L*. The temperature as an ambient factor has a certain effect on the phase. Similarly, the strain also leads to phase change which is related to *L* and *n*. 

The variation of the ambient temperature and strain actually changes the phase of the fiber. Both of them change the phase of the scattering center by affecting the refractive index (*n*) and the length of the fiber (*L*). Correspondingly, the cumulative phase term (Δ*θ*(Δ*s,t*)) is also changed. However, the influence of temperature and strain on the fiber is generally very slight or even absent. It may be more significant when the optical fiber is in a hostile environment.

### 2.3. The Principle of Empirical Mode Decomposition (EMD)

The phase drift *ϕ*_drift_(Δ*s,t*) has the feature of low-frequency and varies with Δ*s*. When the low-frequency external disturbance at the Hz or sub-Hz level occurs, a long detection time is needed to accurately restore the vibration. Thus, the inhibitory effect produced by phase drift will be evident in this case. Furthermore, the phase drift will mix with low-frequency disturbance when their frequency components are very close. In this case, traditional digital filters do not have the ability to separate the phase drift and actual phase from the phase signal after demodulation, even if the order of the filter is very high. Owing to the similar frequency components, wavelet analysis has the problems of a high decomposition layer and difficult wavelet basis selection. So, an algorithm is needed that can be applied for time domain analysis of the signal and is adaptive for different signals. In general, vibration signals have the characteristics of non-linearity, non-stationarity, and rapid change. On account of the above factors, EMD is proposed to eliminate the significant negative effect of phase drift on phase demodulation in the Ф-OTDR system.

**Figure 3:**
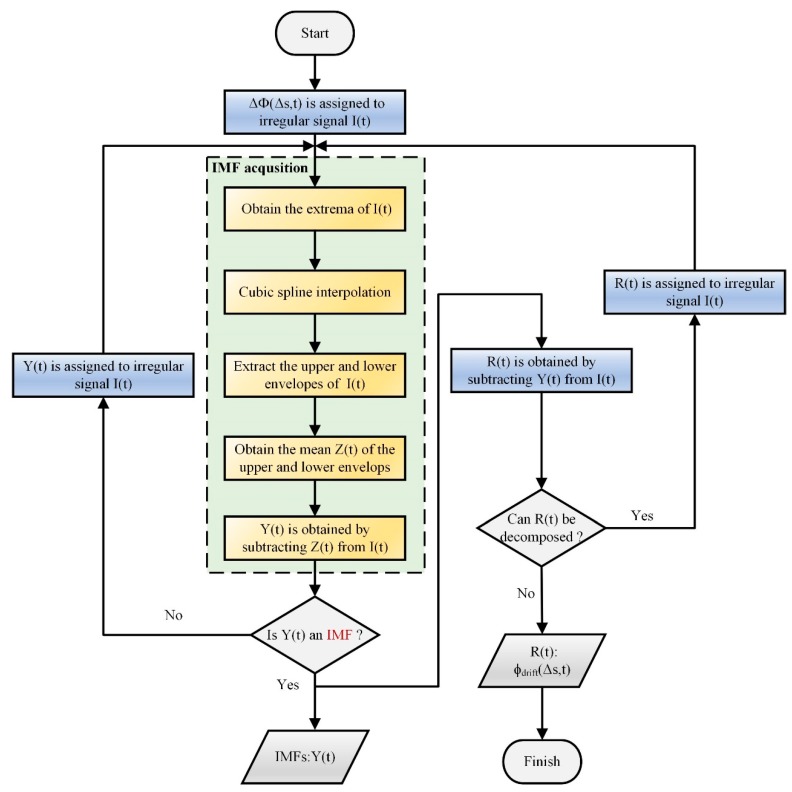
The phase signal Δ*Ф*(Δ*s,t*) decomposition process with the empirical mode decomposition (EMD) algorithm.

EMD is a novel data analysis method and was firstly presented by Huang et al. in 1998 [26]. It is an adaptive data processing algorithm and can realize the decomposition of any type of signal. It decomposes the signal by utilizing the characteristic time scale of the data, and then the baseline is obtained eventually. It is very suitable for analyzing non-linear and non-stationary signals. The decomposition principle of EMD is to decompose a wave with complex frequency components into multiple intrinsic mode functions (IMFs) and one residual wave [27,28,29]. An IMF is a local characteristic signal of the original signal at different time scales and needs to satisfy the following two conditions: (1) The numbers of extrema and zero points are the same. (2) The mean of the upper and lower envelopes is zero. The signal decomposition process using the EMD method is shown in Figure 3 and described as follows.

Firstly, the input signal (*I*(*t*)) is the phase signal Δ*Ф*(Δ*s,t*). All extrema of the *I*(*t*) are calculated, and the values and number of extrema are obtained. Then, the upper and lower envelopes of *I*(*t*) are fitted by a cubic spline interpolation algorithm and are used to calculate their mean, which is denoted *Z*(*t*). A new signal which is denoted *Y*(*t*) is obtained by subtracting *Z*(*t*) from the original signal *I*(*t*). Afterward, we judge whether *Y*(*t*) is an IMF. If *Y*(*t*) is not an IMF, it will be assigned to the irregular signal *I*(*t*) and signal decomposition is executed again until the IMF appears and is output. Otherwise, the difference between *Y*(*t*) and *Z*(*t*) is obtained and called the residual component *R*(*t*). Finally, we judge whether *R*(*t*) can be decomposed. If it can be decomposed, *R*(*t*) is also assigned to the irregular signal *I*(*t*) and signal decomposition is executed again. When the number of either maximum points or minimum points is zero or *R*(*t*) is a monotone function, the data decomposition process is finished. Each IMF and the residual wave are obtained and outputted. The residual wave is the phase drift and the accumulative value of each IMF is the actual phase in the Ф-OTDR system. Consequently, the EMD method can be used to extract the phase drift and actual vibration phase from the phase after demodulation. Perturbation events at the Hz or sub-Hz level can be detected and restored accurately by using this method.

## 3. Experiment Results and Discussions

### 3.1. Validation Experiments of Phase Drift and EMD Method

In our experiments, the repetition frequency and pulse width of the pulsed light were 500 Hz and 150 ns, respectively. The PZT acted at the position of 524 m as the vibration source in a sinusoidal waveform with a frequency of 5 Hz, while the total length of optical fiber was 1038 m. The acquisition rate of the system was 1000 MSa/s, and the detection time was set to 10 s in order to acquire the vibration signal and phase drift.

**Figure 4:**
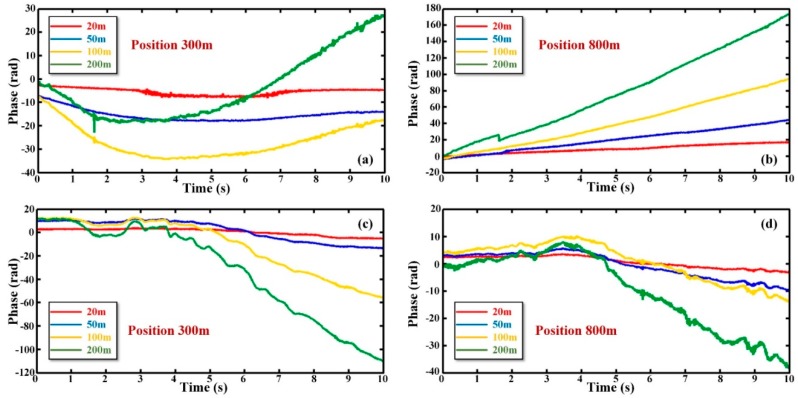
The relationship between phase curves and Δ*s* in the cases of different positions and vibration-free; (**a**) the phase curve when the location is 300 m in the first record; (**b**) the phase curve when the location is 800 m in the first record; (**c**) the phase curve when the location is 300 m in the second record; (**d**) the phase curve when the location is 800 m in the second record.

In order to demonstrate that the phase drift is related to system factors in the condition of isolating ambient temperature and strain, the experiments were carried out and some measures were taken. Better soundproof protection for optical fiber was implemented and the experiments were completed in a room with constant temperature of 25 °C. The experimental justification results are shown in Figure 4 when the selected positions are 300 m and 800 m. Both of them were in a vibration-free environment. The phase traces are displayed when Δ*s* was 20 m, 50 m, 100 m, and 200 m. Figure 4a,b are from the first record. It can be seen that the phase drift still exists when the influences of the ambient temperature and strain have been avoided. Figure 4c,d are from the second record. These two figures also reflect the same rule. From Figure 4, it can be illustrated that the phase drift is related to system factors. When the Δ*s* increases from 20 m to 200 m, the curve fluctuates and the phase drift becomes more deteriorating. This proves that the phase drift increases with Δ*s*, which corresponds to Equation (13).

**Figure 5:**
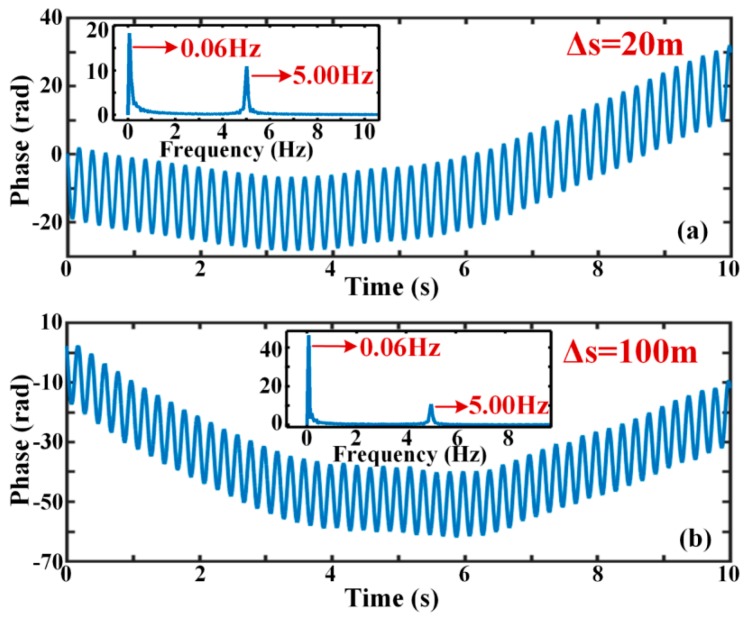
The phase curves in the condition of 5 Hz vibration for different Δ*s;* (**a**) the phase curve and frequency spectrum when Δ*s* is 20 m; (**b**) the phase curve and frequency spectrum when Δ*s* is 100 m.

Figure 5a,b expresses the phase change when the vibration is a sinusoidal signal of 5 Hz and 10 V_pp_. By fast Fourier transform (FFT) of the phase curve, it can be seen that there is not only a 5 Hz vibration signal but also a 0.06 Hz low-frequency component. This low-frequency component is known as the baseline. It has the feature of slow variation and drives the real phase curve to float up and down as a whole. This further proves the existence of low-frequency phase drift. In Figure 5a, the amplitudes of the actual phase and phase drift are about 18.34 dB and 10.91 dB. In Figure 5b, the amplitudes of the actual phase and phase drift are approximately 46.38 dB and 10.69 dB. By comparing Figure 5a with Figure 5b, it can be concluded that the phase drift increases with Δ*s*, but the amplitude of phase induced by external disturbance is roughly the same.

**Figure 6:**
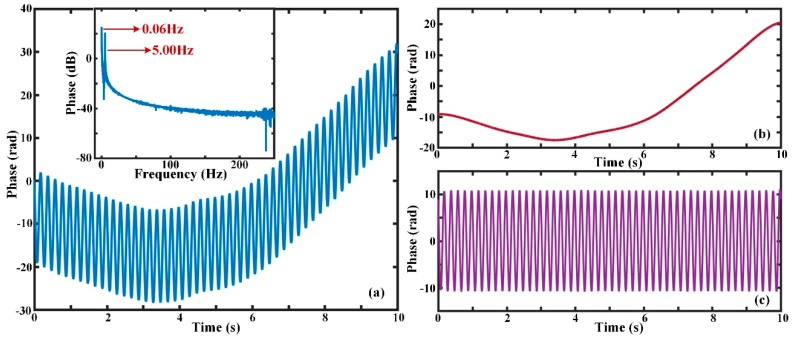
Phase variation curves; (**a**) phase variation after demodulation and its frequency spectrum; (**b**) the phase drift *ϕ*_drift_(Δ*s,t*) obtained by the EMD method; (**c**) the phase signal after phase drift elimination by the EMD method.

Figure 6a indicates the phase change after demodulation when the vibration is a sinusoidal signal with frequency of 5 Hz and intensity of 10 V_PP_, and the Δ*s* is 20 m. It can be seen that the whole fluctuation of the curve is large and reaches about 38.04 rad. By FFT analysis of the phase signal, there was not only a frequency component of 5 Hz caused by external perturbation but also a low-frequency residual wave of 0.06 Hz. Figure 6b,c were obtained when the original phase after demodulation was decomposed and processed by the EMD method.

Figure 6b expresses the residual wave after using EMD decomposition and is a change curve produced by phase drift. Figure 6c represents the actual phase variation under the condition of phase drift elimination. The feasibility and effectiveness of the EMD algorithm for the extraction and elimination of low-frequency phase drift were, thus, verified. Therefore, the EMD method can contribute to the recovery of perturbation events of Hz or sub-Hz level in practice.

### 3.2. Phase Drift Elimination under Different Experiment Conditions

#### 3.2.1. Phase Variation in Different Vibration Frequencies

**Figure 7:**
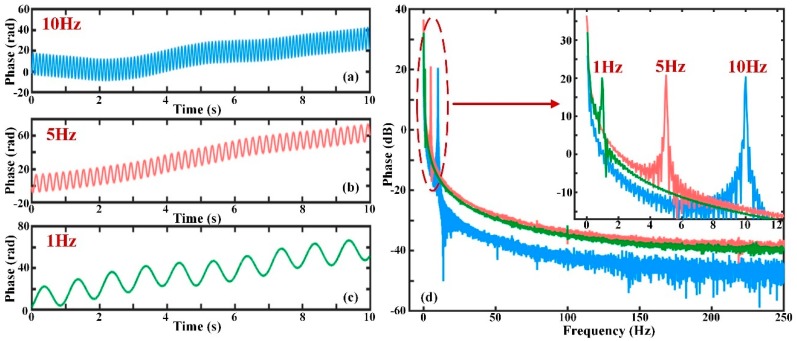
The phase variation with different frequencies and frequency spectrum; (**a**) when the vibration is a sinusoidal signal with frequency of 10 Hz; (**b**) when the vibration is a sinusoidal signal with frequency of 5 Hz; (**c**) when the vibration is a sinusoidal signal with frequency of 1 Hz; (**d**) the fast Fourier transform (FFT) analysis results of phase signals when the vibration frequencies are 10 Hz, 5 Hz, and 1 Hz.

The phase demodulation results when the frequency of perturbation changes and the Δ*s* is 20 m are shown in Figure 7. Figure 7a–c indicate the phase signal after demodulation when the external vibrations are sinusoidal waves with frequency values of 10 Hz, 5 Hz, and 1 Hz, respectively. It can be concluded that the phase drift always exists and is a low-frequency signal. The phase drift causes the whole undulation of the actual phase curve in Figure 7a–c. The FFT results for the three different frequencies are displayed in Figure 7d. All the amplitudes of the actual phases produced by sinusoidal signals at the frequencies of 10 Hz, 5 Hz and 1 Hz are approximately 10 rad (20 dB). However, the amplitude of the phase drift is far greater than that of the vibration; this will significantly affect the recovery of the signal.

**Figure 8:**
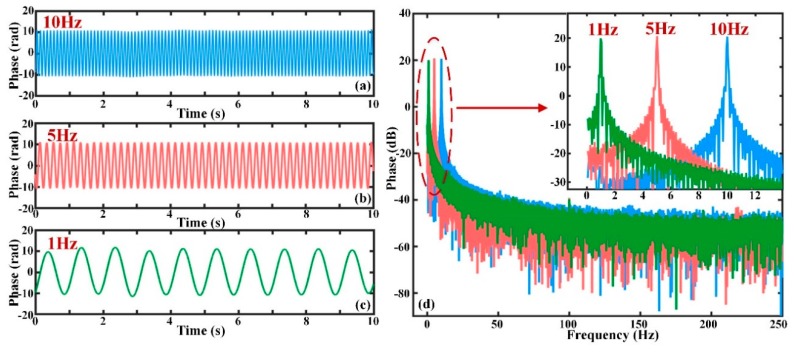
The actual phase variation after phase drift elimination and frequency spectrum (**a**) when the vibration is a sinusoidal signal with frequency of 10 Hz; (**b**) when the vibration is a sinusoidal signal with frequency of 5 Hz; (**c**) when the vibration is a sinusoidal signal with frequency of 1 Hz; (**d**) the FFT analysis results of the actual phase signals when the vibration frequencies are 10 Hz, 5 Hz, and 1 Hz.

The phase change results after using the EMD algorithm are shown in Figure 8a–c. Figure 8a–c indicate the phase signals without phase drift when the external vibrations are sinusoidal waves with frequencies of 10 Hz, 5 Hz, and 1 Hz, respectively. By comparing Figure 7a and Figure 8a, it can be seen that the 10 Hz sinusoidal wave was well restored and the phase drift was successfully eliminated. These comparison results were also seen for the other frequency components of 5 Hz and 1 Hz. From Figure 8d, the amplitude of the actual phase signal was not affected after the low-frequency component was removed. The feasibility and validity of phase drift elimination using the EMD algorithm is, thus, verified when the external disturbance is a sinusoidal signal with different frequencies.

#### 3.2.2. Phase Variation in Different Vibration Intensities

**Figure 9:**
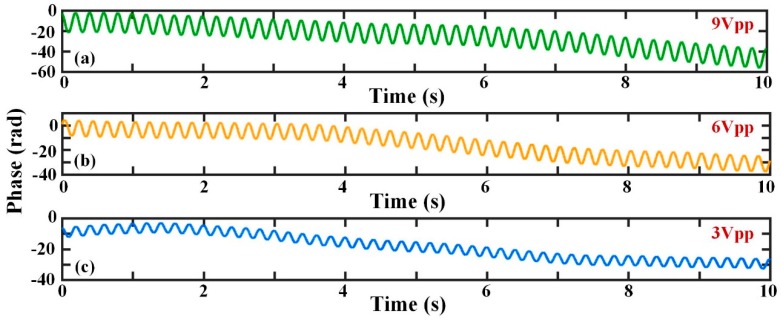
Phase variation after demodulation in the different vibration intensities; (**a**) when the vibration intensity is 9 V_pp_; (**b**) when the vibration intensity is 6 V_pp_; (**c**) when the vibration intensity is 3 V_pp._

**Figure 10:**
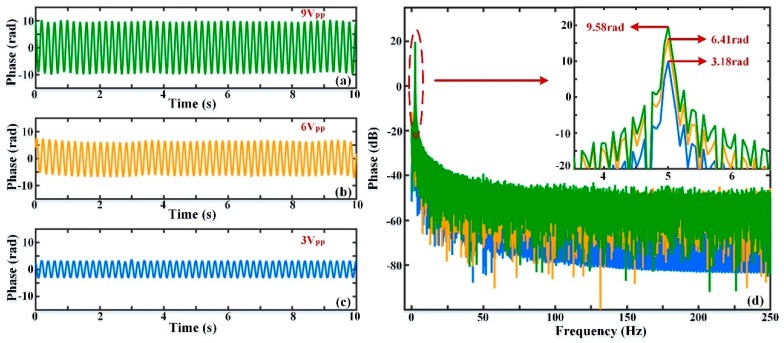
Phase variation after phase drift elimination and frequency spectrum; (**a**) when the vibration intensity is 9 V_pp_; (**b**) when the vibration intensity is 6 V_pp_; (**c**) when the vibration intensity is 3 V_pp_; (**d**) the FFT analysis results of the actual phase signal when the vibration intensities are 9 V_pp_, 6 V_pp_, and 3 V_pp._

The PZT phase modulator acted as the vibration source of sinusoidal form with frequency of 5 Hz, and the Δ*s* value is 20 m. For vibration intensities of 9 V_pp_, 6 V_pp_, 3 V_pp_, the phase curves after demodulation are displayed in Figure 9a–c. The phase drift was always present and badly affected the recovery quality of the vibration signal. Similarly, for vibration intensities of 9 V_pp_, 6 V_pp_, and 3 V_pp_, the actual phase signals after phase drift elimination are displayed in Figure 10a–c. By comparing Figure 9a–c and Figure 10a–c, it is illustrated that the phase drift was extracted and eliminated successfully by using the EMD method, and the amplitude of the phase curves varied with the intensity of the external vibration. In order to represent the correlation between the amplitude of the phase signal and the intensity of vibration, the FFT results of the phase signals are shown in Figure 10d. The amplitudes of the phase signals after FFT analysis were 19.63 dB (9.58 rad), 16.14 dB (6.41 rad), and 10.05 dB (3.18 rad) when the vibration intensities are 9 V_pp_, 6 V_pp_, 3 V_pp_, respectively. This shows that the phase amplitude increases with the vibration intensity when the vibration frequency is the same.

**Figure 11:**
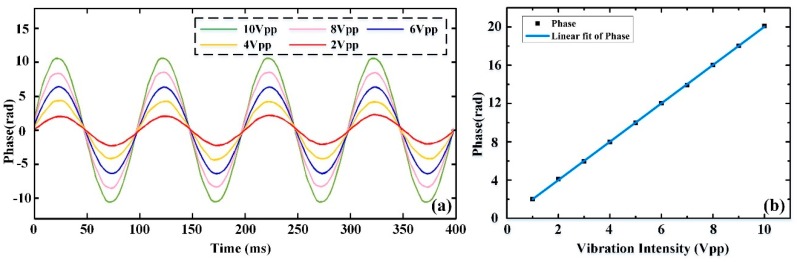
(**a**) Phase variation after phase drift elimination when the vibration intensities are 10 V_pp_, 8 V_pp_, 6 V_pp_, 4 V_pp_, and 2 V_pp_; (**b**) a linear fit curve of actual phase variation.

In order to further verify the relationship between the amplitude of the actual phase signal and the intensity of the external vibration, some experiments were conducted with the intensity of vibration gradually increasing and a Δ*s* value of 20 m. Figure 11a shows the phase recovery results when the vibration was a sinusoidal wave with the frequency of 10 Hz and intensities of 10 V_pp_, 8 V_pp_, 6 V_pp_, 4 V_pp_, and 2 V_pp_. It can be seen clearly that the phase amplitude increased accordingly when the vibration intensity rose. For the sake of better expression of the relationship between the phase amplitude and the vibration intensity, a fit curve was obtained, shown in Figure 11b. The phase amplitude is roughly linearly related to the vibration intensity, and the correlation coefficient is 99.99%.

#### 3.2.3. Phase Variation in Low-Frequency Vibration at the Sub-Hz level

**Figure 12:**
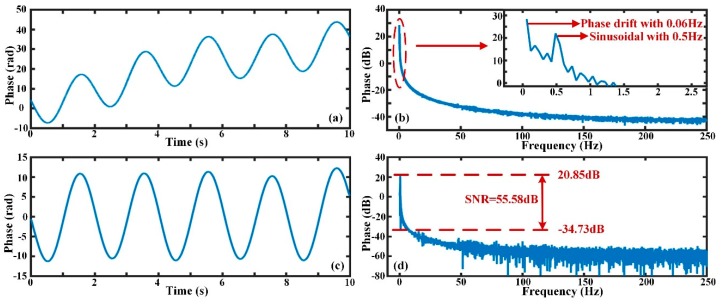
The phase change and frequency spectrum when the vibration frequency is 0.5 Hz; (**a**) the original phase after demodulation; (**b**) the FFT result of the original phase signal; (**c**) the actual phase after phase drift elimination; (**d**) the FFT result and signal-to-noise ratio (SNR) of the actual phase signal.

To test the feasibility and effectiveness of the EMD method in the case of low-frequency external perturbance at the sub-Hz level, sinusoidal signals with different low frequencies were imposed on the PZT. Figure 12 expresses the phase change curves before and after phase drift was eliminated when the exerted vibration was a sinusoidal signal with frequency of 0.5 Hz, intensity of 10 V_pp_ and the Δ*s* value was equal to 20 m. In Figure 12a,b, the phase signal after demodulation includes a sinusoidal signal of 0.5 Hz and a low-frequency component of 0.06 Hz. By executing the EMD algorithm, the phase drift was extracted and eliminated, as shown in Figure 12c,d. From the result after FFT analysis, the amplitude of the sinusoidal signal with frequency 0.5 Hz was approximately 20.85 dB, and the ambient noise level was about −34.73 dB (0.0183 rad/√0.5 Hz = 0.026 rad/√Hz), so the SNR of the phase signal was 55.58 dB.

**Figure 13:**
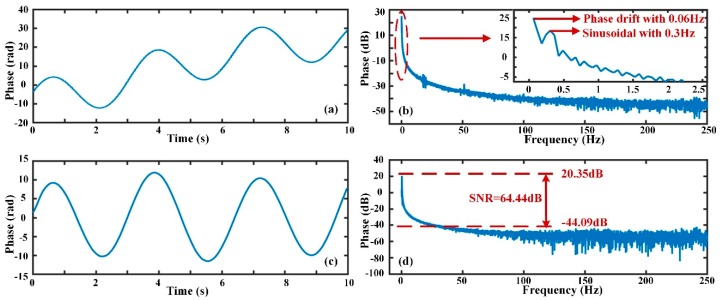
The phase change and frequency spectrum when the vibration frequency is 0.3 Hz; (**a**) the original phase after demodulation; (**b**) the FFT result of the original phase signal; (**c**) the actual phase after phase drift elimination; (**d**) the FFT result and SNR of the actual phase signal.

Similarly, Figure 13 shows the demodulation results and FFT analysis results of the phase signal when a sinusoidal signal occurred with frequency 0.3 Hz, intensity 10 V_pp_ and the Δ*s* was equal to 20 m. From Figure 13a,b, the phase signal was decomposed into a sinusoidal signal of 0.3 Hz and a low-frequency component of 0.06 Hz. The actual phase variation induced by external vibration is illustrated in Figure 13c,d where the phase drift was extracted and eliminated. From Figure 13d, the amplitude of the demodulated 0.3 Hz sinusoidal signal was 20.35 dB, and the background noise was approximately −44.09 dB (0.0062 rad/√0.3 Hz = 0.011 rad/√Hz), so the SNR of the phase signal was 64.44 dB. From Figure 12 and Figure 13, it can be concluded that low-frequency phase drift can be extracted and eliminated by the EMD method, and sub-Hz level sinusoidal signals can be restored. This is beneficial for the restoration of low-frequency disturbance events with sub-Hz level.

## 4. Conclusions

In this paper, we studied phase drift and its elimination in the coherent detection Ф-OTDR system. The correlation between the phase drift and the length of the vibration region was proved in theory. The phase variation deteriorates when the length of the vibration region increases, as demonstrated by the experiments. By analyzing the characteristics of phase drift, the EMD method was proposed to eliminate phase drift and recovery of the vibration signal, especially when the frequency is at the Hz or sub-Hz levels. In the experiments, phase variations induced by sinusoidal signals with different frequencies and different intensities were restored. A linear relationship between the phase and vibration intensity was proved, and the correlation coefficient was 99.99%. In addition, the phase drift was successfully eliminated in the case of low-frequency external vibration of 0.5 Hz or 0.3 Hz. The SNRs of the phase signals after phase drift elimination reached 55.58 dB and 64.44 dB, respectively, in the coherent detection Ф-OTDR system. In conclusion, the EMD algorithm can eliminate phase drift effectively and contribute to the detection and recovery of low-frequency disturbance events at the Hz or sub-Hz level in practical applications.

## References

[1] Dai Y., Sun Q.Z., Tan S.S., Wo J.H., Zhang J.J., Liu D.M. (2012). Highly sensitive liquid-level sensor based on dual-wavelength double-ring fiber laser assisted by beat frequency interrogation. Opt. Express.

[2] He H.J., Shao L.Y., Li Z.L., Zhang Z.Y., Zou X.H., Luo B., Pan W., Yan L.S. (2016). Self-Mixing demodulation for coherent phase-sensitive OTDR system. Sensors.

[3] Hui X.N., Zheng S.L., Zhou J.H., Chi H., Jin X.F., Zhang X.M. (2014). Hilbert-Huang transform time-frequency analysis in phi-OTDR distributed sensor. IEEE Photonics Technol. Lett..

[4] Wang Y., Yuan H.Y., Liu X., Bai Q., Zhang H.J., Gao Y., Jin B.Q. (2019). A Comprehensive Study of Optical Fiber Acoustic Sensing. IEEE Access.

[5] Bao X.Y., Chen L. (2012). Recent progress in distributed fiber optic sensors. Sensors.

[6] Wang Y., Wang P.F., Ding K., Li H., Zhang J.G., Liu X., Bai Q., Wang D., Jin B.Q. (2019). Pattern recognition using relevant vector machine in optical fiber vibration sensing system. IEEE Access.

[7] Tejedor J., Martins H.F., Piote D., Macias-Guarasa J., Pastor-Graells J., Martin-Lopez S., Guillén P.C., Smet F.D., Postvoll W., González-Herráez M. (2016). Toward prevention of pipeline integrity threats using a smart fiber-optic surveillance system. J. Lightwave Technol..

[8] Peng F., Wu H., Jia X.H., Rao Y.J., Wang Z.N., Peng Z.P. (2014). Ultra-long high-sensitivity F-OTDR for high spatial resolution intrusion detection of pipelines. Opt. Express.

[9] Fan X.Y., Yang G.Y., Wang S., Liu Q.W., He Z.Y. (2017). Distributed fiber-optic vibration sensing based on phase extraction from optical reflectometry. J. Lightwave Technol..

[10] Pan Z.Q., Liang K.Z., Zhou J., Ye Q., Cai H.W., Qu R.H. Interference-fading-free phase-demodulated OTDR system. Proceedings of the 22nd International Conference on Optical Fiber Sensors.

[11] Tu G.J., Zhang X.P., Zhang Y.X., Zhu F., Xia L., Nakarmi B. (2015). The Development of an Φ-OTDR System for Quantitative Vibration Measurement. IEEE Photonics Technol. Lett..

[12] Lu Y.L., Zhu T., Chen L., Bao X.Y. (2010). Distributed vibration sensor based on coherent detection of phase-OTDR. J. Lightwave Technol..

[13] Martins H.F., Shi K., Thomsen B.C., Martin-Lopez S., Gonzalez-Herraez M., Savory S.J. (2016). Real time dynamic strain monitoring of optical links using the backreflection of live PSK data. Opt. Express.

[14] Wang Z.N., Zhang L., Wang S., Xue N.T., Peng F., Fan M.Q., Sun W., Qian X.Y., Rao J.R., Rao Y.J. (2016). Coherent Phi-OTDR based on I/Q demodulation and homodyne detection. Opt. Express.

[15] Pan Z.Q., Liang K.Z., Ye Q., Cai H.W., Qu R.H., Fang Z.J. Phase-sensitive OTDR system based on digital coherent detection. Proceedings of the Conference on Optical Sensors and Biophotonics III.

[16] Qin Z.G., Zhu T., Chen L., Bao X.Y. (2011). High sensitivity distributed vibration sensor based on polarization-maintaining configurations of phase-OTDR. IEEE Photonics Technol. Lett..

[17] Lindsey N.J., Martin E.R., Dreger D.S., Freifeld B., Cole S., James S.R., Biondi B.L., Ajo-Franklin J.B. (2017). Fiber-optic network observations of earthquake wavefields. Geophys. Res. Lett..

[18] Yuan Q., Wang F., Liu T., Liu Y., Zhang Y.X., Zhong Z., Zhang X.P. (2019). Compensating for influence of laser frequency-drift in phase-sensitive OTDR with twice differential method. Opt. Express.

[19] Healey P. (1987). Statistics of Rayleigh backscatter from a single-mode fiber. IEEE Trans. Commun..

[20] Zhu F., Zhang X.P., Xia L., Guo Z., Zhang Y.X. (2015). Active compensation method for light source frequency drifting in Ф-OTDR sensing system. IEEE Photonics Technol. Lett..

[21] Fernández-Ruiz M.R., Pastor-Graells J., Martins H.F., Garcia-Ruiz A., Martin-Lopez S., Gonzalez-Herraez H. (2018). Laser phase-noise cancellation in chirped-pulse distributed acoustic sensors. J. Lightwave Technol..

[22] Xue N.T., Fu Y., Lu C.Y., Xiong J., Yang L., Wang Z.N. (2018). Characterization and compensation of phase offset in Φ-OTDR with heterodyne detection. J. Lightwave Technol..

[23] Wu M.S., Fan X.Y., Liu Q.W., He Z.Y. (2018). Highly sensitive quasi-distributed fiber-optic acoustic sensing system by interrogating a weak reflector array. Opt. Lett..

[24] Park J., Taylor H.F. (2003). Fiber optic intrusion sensor using coherent optical time domain reflectometer. Jpn. J. Appl. Phys..

[25] Sha Z., Feng H., Zeng Z.M. (2017). Phase demodulation method in phase-sensitive OTDR without coherent detection. Opt. Express.

[26] Huang N.E., Shen Z., Long S.R., Wu M.C., Shih H.H., Zheng Q., Yen N.C., Tung C.C., Liu H.H. (1998). The empirical mode decomposition and the Hilbert spectrum for nonlinear and non-stationary time series analysis. Proc. R. Soc. Lond. A.

[27] Boudraa A.O., Cexus L.C., Saidi Z. (2004). EMD-Based signal noise reduction. Int. J. Signal Process.

[28] Omitaomu O.A., Protopopescu V.A., Ganguly A.R. (2011). Empirical mode decomposition technique with conditional mutual information for denoising operational sensor data. IEEE Sens. J..

[29] Hassan M., Boudaoud S., Terrien J., Karlsson B., Marque C. (2011). Combination of canonical correlation analysis and empirical mode decomposition applied to denoising the labor electrohysterogram. IEEE Trans. Biomed. Eng..

